# Prehospital stroke diagnostics using three different simulation methods: A pragmatic pilot study

**DOI:** 10.1177/23969873241252564

**Published:** 2024-05-16

**Authors:** Emma Christensen, Helge Fagerheim Bugge, Jostein Hagemo, Karianne Larsen, Astrid KV Harring, Jostein Gleditsch, Jørgen Ibsen, Mona Guterud, Else Charlotte Sandset, Maren Ranhoff Hov

**Affiliations:** 1 Institute of Clinical Medicine, Faculty of Medicine, University of Oslo, Oslo, Norway; 2 Department of Research, Norwegian Air Ambulance Foundation, Norway; 3 Department of Neurology, Oslo University Hospital, Oslo, Norway; 4 Department of Health Science, Oslo Metropolitan University, Oslo, Norway; 5 Department of Radiology, Østfold Hospital, Sarpsborg, Norway; 6 Department of Medicine, Ringerike Hospital, Vestre Viken Hospital Trust, Honefoss, Norway; 7 Division of Prehospital Services, Oslo University Hospital, Oslo, Norway

**Keywords:** Prehospital, CT, MSU, helicopter, rural

## Abstract

**Introduction:**

The optimal pathway for ultra-early diagnostics and treatment in patients with acute stroke remains uncertain. The aim of this study was to investigate how three different methods of simulated, rural prehospital computed tomography (CT) affected the time to prehospital treatment decision in acute stroke.

**Materials and Methods:**

In this pragmatic, simulation, pilot study of prehospital CT we investigated a conventional ambulance with transport to a standard care rural stationary CT machine managed by paramedics, a Mobile Stroke Unit (MSU), and a helicopter with a simulated CT machine. Each modality completed 20 real-life dispatches combined with simulation of predetermined animated patient cases with acute stroke symptoms and CT images. The primary endpoint of the study was the time from alarm to treatment decision.

**Results:**

Median time from alarm to the treatment decision differed significantly between the three groups (*p* = 0.0005), with 38 min for rural CT, 33 min for the MSU, and 30 min for the helicopter. There was no difference in time when comparing rural CT with MSU, nor when comparing the MSU with the helicopter. There was a difference in time to treatment decision between the rural CT and the helicopter (*p* < 0.0001). The helicopter had significantly lower estimated time from treatment decision to hospital (*p* = 0.001).

**Disscussion/Conclusion:**

Prehospital CT can be organized in several ways depending on geography, resources and need. Further research on paramedic run rural CT, MSU in rural areas, and helicopter CT is needed to find the optimal strategy.

## Graphical abstract

**Graphical abstract:**
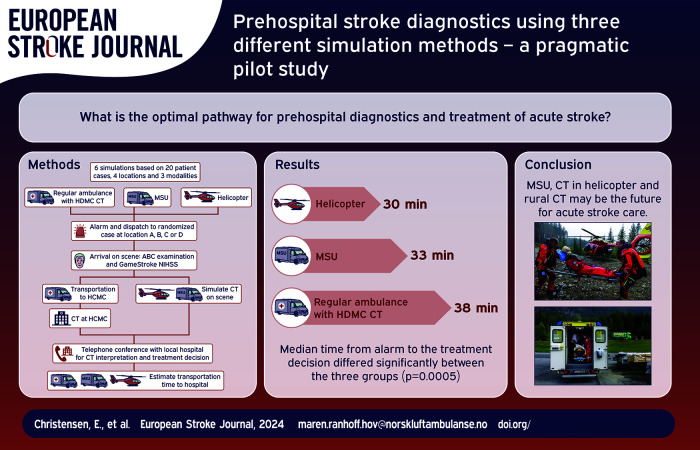

## Background

Prehospital delay represents a significant part of the total delay to therapy in acute stroke.^1^ Increased availability of computed tomography (CT) in the prehospital field, especially in rural areas, can potentially reduce the time to diagnosis, treatment, and help limit transportation delays in stroke patients.

The most common pathway for hospital admittance of stroke patients in Norway is by ambulance. The Norwegian emergency medical service (EMS) is government-funded, and the ambulances are staffed with a 2-person crew.^2,3^ The ambulance crew consist of emergency medical technicians and paramedics where some have additional training as nurses,^2^ for simplicity hereafter called paramedics.

Paramedics dispatched to a patient with a suspected acute stroke will screen for stroke symptoms by using Face Arm Speech Test (FAST) or National Institutes of Health Stroke Scale (NIHSS).^4^ Patients with suspected stroke will be brought to hospital for a CT examination after pre-notification of the on-call stroke physician.

Several models of prehospital stroke assessment have been tested and implemented over the past decade.^5–8^ No consensus of the optimal handling of patients with suspected acute stroke in the prehospital setting has been reached.^9,10^ In recent years several studies have been conducted in Norway in an attempt to increase skill and diagnostic accuracy among prehospital personnel, and to increase the availability of prehospital CT.^5,11–13^

In the rural prehospital CT model at Ål, Norway, paramedics are trained to perform CT scan at Hallingdal District Medical Center (HDMC), a rural central with out of hours general practitioner (GP) and radiological unit. The CT scanner is currently standard care after evaluation in a clinical study, and all patients with a suspect stroke in the catchment area are brought in for examination. The scanner is used in acute settings like stroke, as well as elective controls. All CT examinations are assessed via telemedicine to the local hospital at Ringerike Hospital. In case of acute stroke, paramedics are trained to conduct the CT using telemedicine and if indicated, initiate thrombolytic treatment as a standard prehospital treatment option.^14^ The clinical study of this model showed a significant reduction in time to thrombolytic therapy compared to transportation and in-hospital assessment at the local hospital (Ibsen et al, unpublished). This new prehospital CT concept is inspired by the Mobile stroke units (MSU) that over recent years have been validated and recommended in European Stroke Organization guidelines.^15^

Both the MSU and the rural CT apply a CT scanner that allows for prehospital diagnosis and initiation of treatment. In the Norwegian TreatNASPP trial, the MSU was staffed with prehospital personnel trained to conduct CT examinations and initiate thrombolytic therapy prehospital. The study found a significantly reduced time from alarm to thrombolysis and increased the number of patients who receive treatment.^11,15^ Though the MSU model is potentially cost-effective in densely populated areas,^16,17^ there is scarce information on the general efficiency of MSUs in rural areas.^18,19^

In sparsely populated areas, with challenging infrastructure, the helicopter emergency medical service (HEMS) plays an important role to provide critical care for all. The Norwegian HEMS is a nationwide service consisting of 13 bases. Each helicopter is operated by a team consisting of one pilot, one rescue paramedic, and one anesthesiologist.^20^ Currently, there are no helicopters with the capability of cerebral imaging, but a helicopter equipped with a CT would strongly reduce time to diagnostics in areas with long transportation distances.^18^ Research on prehospital stroke treatment has sought to develop the prehospital diagnosis of stroke in the HEMS, as well as the establishment of CT in helicopters.^18^ This research is still at a theoretical level and will need further development of lightweight CT scanners, and exploratory and implementational research to determine whether helicopter CT can reduce the time from alarm to stroke diagnostics and treatment for patients in rural areas.

In this pragmatic simulation-based pilot study, the aim was to explore the feasibility of three novel methods of rural, prehospital CT diagnostics, focusing on the time spent from alarm to treatment and destination decisions in pre-developed scenarios of acute stroke assessment. We used regular ambulance services combined with the standard care rural CT in HDMC at Ål, an MSU, and an air ambulance helicopter to perform the study.

## Methods

### Location and study design

This was a pragmatic prehospital simulation study comparing three different methods of prehospital CT for stroke patients in a rural setting. The study was conducted from June 19th, 2023 to June 22nd, 2023 in Hallingdal, Norway. Hallingdal is a rural valley area located in southern Norway. It covers an area of 5820 km^2^, with approximately 20,000 inhabitants. HDMC is in Ål, the largest of six municipalities with its 4600 people. The entire Hallingdal valley is part of Vestre Viken Hospital Trust, and the closest hospital is Ringerike hospital, 141 km and an approximately 2-h drive from Ål. Ringerike hospital is a primary stroke center managed by specialist in internal and geriatric medicine. The secondary stroke center is located at Oslo University Hospital, Rikshospitalet with a driving distance of 193 km from HDMC and 54 km from Ringerike hospital. The HDMC provides specialist healthcare to Hallingdal through a somatic in-patient ward, a radiology clinic including a stationary CT scanner, and an out-patients clinic. The prehospital services in Ål are located in the same building. To become an ambulance personnel in Norway, there are different pathways. In the high school system, there is an emergency medical technician level program with 2 years of theoretical education followed by a 2-year apprenticeship within the ambulance service. Training for a paramedic level is possible for emergency medical technicians (EMTs) and for registered nurses through additional education within the college and university system. From 2014 a bachelor’s program for paramedicine as well as a master’s program in prehospital critical care are embedded in the Norwegian education system. Norwegian ambulance crews consist of paramedics, EMTs or registered nurses with customized training. For simplicity, any ambulance personnel are hereafter called paramedics.

**Figure 1.**
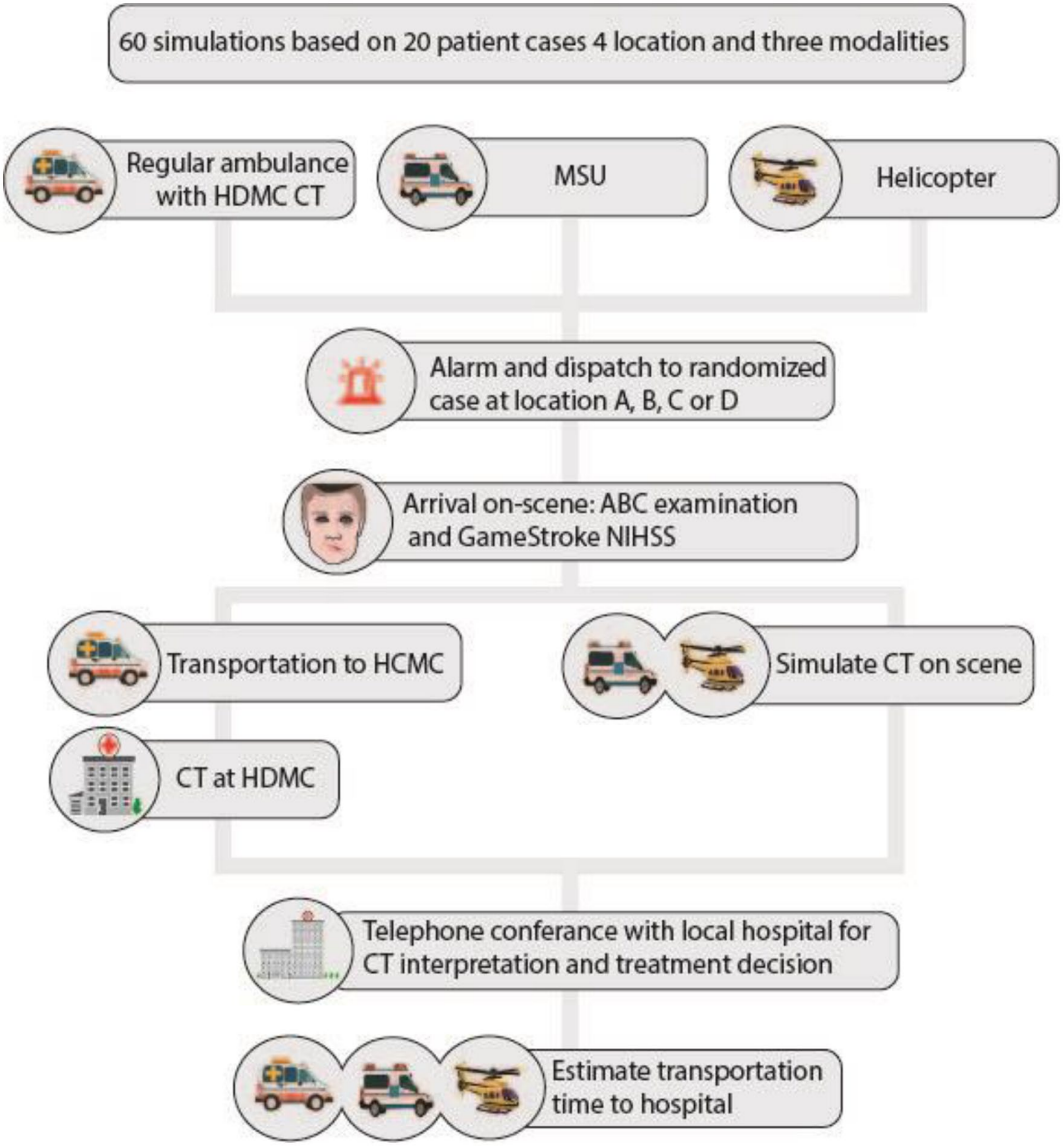
Flow chart of the three study arms. The three arms of the study from dispatch to treatment decision and selection of hospital for final treatment.

Three different prehospital resources were dispatched to simulated patients with suspected acute stroke to evaluate different methods of prehospital CT in a rural setting (Figure 1). The first of the three arms in this study was a conventional ambulance, staffed with two paramedics with access and training in the use of the rural CT machine at HDMC and clinical assessment of NIHSS. The second arm was an MSU, staffed with two paramedics trained for operating the MSU including CT scan assessment, NIHSS and initiation of thrombolytics. The third arm of the study was a helicopter staffed like the Norwegian HEMS, with both a paramedic and anesthesiologist trained in acute stroke assessment like in the MSU. Figure 1 shows a flowchart of the study and the three arms. All participants volunteered and received a salary compensation for participation.

**Figure 2.**
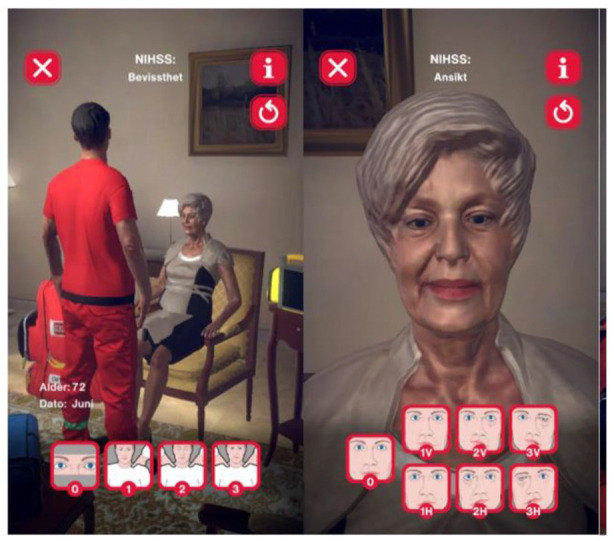
GameSTROKE illustration. Two screenshots from GameSTROKE. On the right is an example of NIHSS 1a Level of Consciousness. On the left is an example from NIHSS 4 Facial Palsy.

No real patients were involved. Simulation was done on a dummy patient and through gamification on an iPAD with the specially developed GameSTROKE (Figure 2).^21^

The game contains a 2D animated patient with pre-programed stroke symptoms, and a NIHSS examination based on the recently published ParaNASPP study.^5^ The dummy patient was used to make loading, unloading, and measurements of vital parameters more realistic. GameSTROKE was used to perform prehospital NIHSS and simulate a CT scan. For this study 20 unique cases with a predetermined NIHSS score and corresponding CT scans were made. The CT scans were acquired from the TreatNASPP MSU trial database and matched with cases with similar total NIHSS score. Each of the three arms conducted all 20 cases, meaning a total of 60 simulations were completed (Table 1).

**Table 1.** Overview of simulation locations.

Simulation location	Kilometers from NAAF location by air	Kilometers from NAAF location by road	Number of cases
A	5.6	10.1	4
B	14.8	17.7	8
C	24.1	35.6	7
D	35.2	35.6	1

The four chosen simulation locations, their distance from the NAAF location in nautical miles and kilometers, and the number of simulation located at each location.

The study was conducted from the Norwegian Air Ambulance Foundation (NAAF) training location at Torpomoen, Hallingdal near HMDC. Localization was chosen by pragmatic reasons, due to the access of both the rural CT at HMDC and a HEMS base in close proximity. To illustrate the real-life distribution of stroke incidence in Hallingdal data from the Norwegian stroke registry was used to calculate 20 distances from the NAAF training location to real-life stroke patients. The pre-prepared scenarios had a severity range of NIHSS 0–31 and the cases were randomly assigned with the 20 cases and 4 simulation locations were chosen, based on distance (Table 1, A–D). This was done to ensure that no real-life stroke patients would be identified based on this study, to have suitable landing places for the helicopter, and to minimize any impact from this study on the local population in general.

### Scenarios and simulation

All scenarios were made by programing a complete NIHSS score into the GameSTROKE application and finding suitable CT images from patients with similar symptoms and NIHSS scores. All the CT scans that were used were originally taken in an MSU as part of the TreatNASPP study. Each of these predefined cases were randomly paired with an address and thereby one of our four discharge locations. Vital parameters, a medical history and an ambulance dispatch was supplemented by the research team to make each case as realistic as possible. See Supplemental Material for a complete list of cases, locations, NIHSS scores, CT imaging results and dispatch reason.

In all three arms, the scenarios started with an alarm at the NAAF location followed by a real emergency dispatch of ambulance, MSU or helicopter to one of the four simulation locations. In real-life missions the HEMS crew would have a meeting to plan the mission regarding weather conditions, landing opportunities and general safety precautions. While on scene this was deemed to not be realistic in our approach with four pre-selected landing places and equal weather for consecutive cases. The median time from alarm to takeoff at Ål HEMS base was added to the mission time in the helicopter arm.

Information about previous medical history, time of symptom onset and the patient’s medication were available digitally. Since the arrival time was unknown and different for each simulation, vital parameters were given as a note to read after examination of the dummy patient. Upon arrival on-scene a complete examination was simulated including ABC examination, vital parameters, and clinical stroke assessment with NIHSS. In the rural CT arm the dummy patient was transported to HDMC to perform a simulated CT scan, while in the MSU and the helicopter arms CT scans were simulated on-scene. A phone conference with the stroke team at the local hospital was made, and the stroke physician reviewed the CT scan. CT interpretation was focused on identification of radiological contraindication of thrombolysis.^9^ The results from the medical history, examination and the CT interpretation were the basis for a treatment decision and a decision of which hospital to admit the patient to. The final treatment decision was made after consulting the on-call stroke physician at Ringerike hospital. Before the scenario was finished each crew estimated the time needed for transportation to the hospital chosen for admittance. This estimation was based on experience from the local paramedics and a combination of speed and nautical miles for the helicopter.

### Ethics

No real patients were participating in this study. Data Protection Officers at Vestre Viken approved the use of personnel information for ambulance personnel from Vestre Viken.

### Randomization and masking

The order of the simulations in the three study arms were randomized by an external research colleague, otherwise not involved in this project, using R software. The study participants were blinded to the order, symptoms, diagnosis, and location of the dispatch up to the scenario was live. For administrative purposes this was not blinded from the research team.

### Statistical analysis

Continuous data are reported as mean (standard deviation) while median (inter quartile range) is used for skewed data. Categorical data is presented as absolute numbers and percentages. Two-sided *p*-values will be reported and considered statistically significant if <0.05.

The primary endpoint was time elapsed from alarm to a prehospital treatment decision was made. Time of treatment decision was defined as the time when a stroke physician and the prehospital personnel decide on whether to give prehospital thrombolysis or not, and where the patients should be transported for hospital admittance. Secondary endpoints were time spent on-scene and time from alarm to estimated arrival at hospital. Kruskal-Wallis test was used to compare all groups, and Mann-Whitney *U*-test when comparing one-and-one groups. We corrected for multiple comparisons with Bonferroni correction (dividing the *p*-value (0.05) by the number of comparisons made), *p*-value will be considered significant if it is below 0.017.

All statistical analysis will be done using Stata version 18. EC, HFB and MRH had full access to the data. HFB takes responsibility for data integrity and analysis.

## Results

For the main endpoint there was a significant difference in time from alarm to treatment decision between the three groups (*p* = 0.0005). Median alarm to treatment decision time for regular ambulance, including transport to the HDMC for a CT scan was 38 min. For the MSU and the helicopter alarm to treatment decision time was 33 and 30 min, respectively. When directly compared, there was no difference between the regular ambulance and MSU (*p* = 0.05), nor between the MSU and the helicopter (*p* = 0.3). However, there was a difference between the regular ambulance and the helicopter (*p* < 0.0001).

The distances from base to the simulation locations is shown in Table 1. Mean distance from base to simulation was 17.2 km. Median NIHSS of the predefined patient cases was 5.5 (range 0–31). All three study arms conducted all 20 cases.

**Figure 3.**
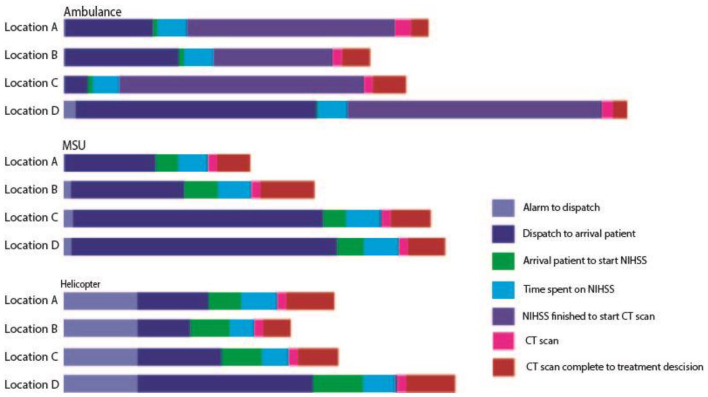
Overview of each time interval from alarm to treatment decision by study arm. The time spent on each interval from alarm to treatment decision is made. Each segment is median time in minutes for a resource at a given simulation location. This illustrates where each resource spends most of the time. Location A is the shortest distance and location D is furthest away.

On-scene time was 6.6 min for the regular ambulance, which was significantly faster than the helicopter crew (14.4 min) and MSU (15.1 min) (*p* = 0.0001). Estimated time from the decision of where to transport the patient until arrival at said location was significantly shorter for the helicopter with a median of 35 min compared with 120 min for both the MSU and the regular ambulance (*p* = 0.0001). For the entire mission, alarm to estimated hospital arrival, there was also a significant time difference between the groups (*p* = 0.0001), with the helicopter being the fastest with 64 min compared to 152 and 153 min in the MSU and regular ambulance (Table 2 and Figure 3).

**Table 2.** Time variables.

	Ambulance with CT at HDMC	MSU	Helicopter
Alarm to dispatch	0.2 (0.1–0.1)	0.9 (0.2–1.2)	8.6 (8.4–8.6)
Dispatch to arrival on-scene	11.3 (3.2–12.9)	14.6 (12.7–29.3)	8.1 (6.0–10.6)
On-scene time^b^	6.6 (5.5–9.2)	15.1 (13.3–16.1)	14.4 (12.7–15.4)
Arrival on-scene to start NIHSS	0.6 (0.4–0.7)	3.2 (2.1–4.4)	4.5 (3.7–5.1)
Time to complete NIHSS	3.5 (3.3–3.7)	3.5 (3.3–4.0)	3.0 (2.8–3.4)
NIHSS finished to start CT	23.6 (15.0–27.2)	0.1 (0.1–0.3)	0.0 (0.0–0.1)
Time CT scan	1.2 (1.1–1.8)	1.1 (1.0–1.1)	1.0 (1.0–1.1)
CT finished to treatment decision	2.7 (1.8–4.1)	4.9 (4.2–5.9)	4.4 (3.2–5.5)
Estimated time to hospital	120.0 (90.0–120.0)	120.0 (92.5–130.0)	35.0 (33.5–40.0)
Alarm to treatment decision^a^	38.1 (36.2–44.3)	33.6 (27.6–43.5)	29.8 (27.0–33.5)
Arrival patient to start CT	28.1 (19.0–30.8)	7.3 (5.8–8.6)	7.7 (6.8–8.5)
Alarm to estimated time for arrival hospital^b^	153.6 (125.4–158.1)	152.9 (120.3–171.5)	64.2 (62.0–72.4)

Time in minutes for intervals between alarm and estimated arrival hospital provided as median (IQR).

a Primary endpoint.

b Secondary endpoints.

## Discussion

This study explores different models for implementation of prehospital CT in rural areas for assessment of suspected stroke patients. In this simulation study we found a significant difference in time from alarm to treatment decision comparing conventional ambulance with rural CT, an MSU and the HEMS, with the most time effective simulation being the helicopter with a CT scanner.

Helicopters equipped with a CT scanner have the potential to be a future time efficient model for acute stroke diagnostics and treatment. Helicopters with CT enables a mobile stroke service that covers long distances and challenging terrains which can be beneficial in areas with little to no-infrastructure. In this study we found that a CT equipped helicopter would reduce the time to treatment decision compared to standard care and we estimated a reduction in time to hospital arrival of more than an hour. This may benefit stroke patients eligible for thrombectomy or in need of neurosurgery without going to the expense of thrombolysis. A Danish simulation study explored the use of helicopters for transportation in a “drip and ship” model or “bypass” model and compared data to conventional ambulance transportation to a comprehensive stroke center for patients with a large vessel occlusion, and showed that helicopter transportation markedly reduced time to thrombectomy.^22^ On the other hand, CT in helicopters may be a vulnerable resource in unstable weather conditions, and on average 13.3% of missions are canceled due to weather conditions in southern Norway. During winter, this number increases to 22.7%.^23^

In a newly published meta-analysis HEMS assessment was not shown to effect mortality but significantly affect neurological outcome in acute stroke.^24^ However, to optimize the helicopter CT model, there could be a stroke severity threshold based on prehospital NIHSS for dispatch and also consider dispatch to other medical and traumatic conditions that could potentially benefit from CT diagnostics. The air ambulance helicopter has a crucial role in the handling of critical medical conditions in the prehospital field and introducing helicopters with CT must be explored with a cautious approach.

Placing the only locally available CT scanner in a helicopter, may cause uncertainty to its actual availability among local ambulance crews. The helicopters, like the MSU, cannot be used as out-patient radiology clinics when idle. This is a clear benefit of the rural stationary CT model. Stationary prehospital CT in rural areas may be remotely controlled and staffed by paramedics during out-of-hours. Recent MSU studies have shown both efficiency and cost benefit in suburban and urban areas.^16,17,25^ However, in rural areas the number of suspected stroke patients are low and most likely not cost beneficial if the CT were limited to this patient group. A recent study, not yet published, exploring prehospital stroke treatment at HMDC show that vast delays in time to revascularization may be avoided with rural CT models, and by allowing other non-emergency patients in need of a CT examination get access the model might be cost-efficient and analysis will be published later.

Even though a rural CT is closer to the patients, they still have to be transported to the CT location. And this is illustrated in our study, by the significantly increased time from arrival at patient location to CT for the regular ambulance (Table 2).

Although MSU-based CT is promising for bringing CT out to the patient, there are limitations that have not been resolved. In the present study, the MSU is the slowest from dispatch to arrival compared to the other simulation arms. One factor that may have contributed is that it is a heavy and large car, leading to challenges in areas with poor road infrastructure. Because of the CT and the total weight of the MSU, it is also very expensive to operate, and might not be suitable for hilly, mountainous, and potentially snowy areas. On the other hand, a health economic analysis shows that MSU care in the Norwegian government funded health care system, can be cost-effective in areas where between 125 and to 260 patients can receive treatment per MSU per year.^16^ New analyses are needed for other models like rural CT and HEMS CT.

In this study we focused on time to treatment decision in suspected stroke, and implementation of prehospital CT in rural areas may possibly reduce onset-to-treatment time and facilitate correct triage and thereby reduce transportation times for stroke patients. It may also reduce transportation times and costs for out-patient appointments, provide relief for the hospital radiology departments, and be of use in other acute medical conditions. A broader use should be explored in further studies.

Rural areas consist of diverse landscapes and may have an impact on the difficulty of the mission. The landscape will probably make a difference in determining which of the modalities is best suited for that assignment, based on where in the rural area the patient is located. Our results may have been different had we chosen another location.

Future prehospital CT models should seek a combination of rural availability, efficient transportation, and access to all stroke patients. The most likely is a scenario where local regular ambulances assess patients before a rendezvous with available resources, either in form of MSU, helicopter or rural CT. For patients that are potential candidates for thrombectomy, HEMS CT and transportation might be the best option, while for patients with subtle symptoms, MSU or rural CT might be the best option. Another plausible scenario is that new technology, prehospital CT scanners will become cheap and available in most ambulances.^23,26^ This would be a gamechanger for prehospital treatment and triage, where paramedics trained in both stroke assessment with NIHSS and performing CT scans will be the future of stroke care in rural areas.

### Limitations

The main limitation of this study is that no helicopters in the Norwegian HEMS is equipped with a CT. Therefore, this study is conducted as a pilot simulation study.

For real-life helicopter missions there is an operational planning meeting before take-off. In this meeting the weather charts are consulted, a suitable landing ground is found, and the technical aspects of the mission is discussed among the crew. In our pre-defined scenarios, all of which were consecutively conducted, this would not accurately reflect reality since it would be the same weather and same landing places for most cases. Also, the strain on turning the helicopter engine on and off 20 times was considered an obstacle for these planning meetings. Therefore, the average real-life time from alarm-to-take-off at Torpomoen Helicopter base was added to time interval between alarm and dispatch retrospectively.

An on-call, operative, helicopter is located at Torpomoen Helicopter. Due to the rest times for this crew the study helicopter could not land at the base one morning. This affected 8 of 20 missions where instead of landing, they remained airborne above the landing site. This was remedied with the crew adding a time delay and registering estimated time spent for landing and take-off.

In our scenarios the helicopter landed close to the patient, while in many real-life missions the HEMS crew requires support from local ambulances to transport the patients to the landing space.

Regular ambulances are a more readily available resource than both MSUs and helicopters, and the closest available resource would be dispatched. To reflect this, the ordinary ambulance crew had two different starting positions in the present study.

In this simulation study the paramedics and prehospital physician involved knew it was a stable stroke patient, and NIHSS was initiated earlier and not secondary to a complete A–E examination. This may have affected time stamps in the study, and highlight the need of a full scale prehospital clinical study in the future.

## Conclusions

Prehospital CT might be the future in acute stroke care, and we need to explore new models to utilize both available prehospital resources and costs. By simulating three different models of prehospital CT we found that rural CT, MSU and HEMS CT may be feasible and could make revascularization available for more people living in rural areas. CT in HEMS is the most effective and development of new technology and clinical studies are needed in the future.

## Supplemental Material

sj-docx-1-eso-10.1177_23969873241252564 – Supplemental material for Prehospital stroke diagnostics using three different simulation methods: A pragmatic pilot study Supplemental material, sj-docx-1-eso-10.1177_23969873241252564 for Prehospital stroke diagnostics using three different simulation methods: A pragmatic pilot study by Emma Christensen, Helge Fagerheim Bugge, Jostein Hagemo, Karianne Larsen, Astrid KV Harring, Jostein Gleditsch, Jørgen Ibsen, Mona Guterud, Else Charlotte Sandset and Maren Ranhoff Hov in European Stroke Journal
