# Supplementary material for: Prehospital stroke diagnostics using three different simulation methods: A pragmatic pilot study
Source: Eur Stroke J. 2024 May 16;9(4):1016–24. doi: 10.1177/23969873241252564 (PMC11569525; doi:10.1177/23969873241252564)
Supplement: sj-docx-1-eso-10.1177_23969873241252564 – Supplemental material for Prehospital stroke diagnostics using three different simulation methods: A pragmatic pilot study [file sj-docx-1-eso-10.1177_23969873241252564.docx]

| **Case** | **Location (A-D)** | **NIHSS score** | **Ischemic or ICH** | **Dispatch**  **reason** |
| --- | --- | --- | --- | --- |
| 1 | B | 6 | Ischemic | Reduced strength on the  left side |
| 2 | B | 5 | Ischemic | Coordination difficulties  right arm |
| 3 | B | 8 | ICH | Paralysis on  the left side |
| 4 | A | 4 | Ischemic | Confusion with  possible visual impairment |
| 5 | C | 20 | Ischemic | Sudden fall at  home |
| 6 | C | 2 | Ischemic | Paralysis left  arm |
| 7 | C | 9 | ICH/SDH | Suddenly confused, accompanied  by headache |
| 8 | A | 3 | Ischemic | Suddenly dizzy |
| 9 | B | 5 | Ischemic | Paralysis on  the right side |
| 10 | B | 7 | Ischemic | Paralysis on the right side, increasing  confusion |
| 11 | B | 6 | Ischemic | Motor  weakness leg |
| 12 | D | 11 | Ischemic | Unclear speech |
| 13 | B | 0 | Ischemic | Sudden dizziness, headache,  vomiting |
| 14 | C | 16 | ICH | Facial paralysis,  rigid gaze to the right side |
| 15 | C | 1 | Ischemic | Sudden loss of  vision |
| 16 | A | 31 | Ischemic | Rigid gaze to  the right |
| 17 | B | 8 | Ischemic | Motor weakness left  arm and leg |
| 18 | C | 10 | Ischemic | Progressive paralysis on the left side,  palpitations |
| 19 | C | 7 | Ischemic | Numbness  right arm, |

|  |  |  |  | possible visual  impairment |
| --- | --- | --- | --- | --- |
| 20 | A | 7 | Ischemic | Acute unclear  speech |
